# Comparison between DSQIID total / sub-item scores and plasma p-tau elevation in adults with Down’s syndrome

**DOI:** 10.1371/journal.pone.0311878

**Published:** 2024-12-09

**Authors:** Makiko Shinomoto, Chisen Takeuchi, Harutsugu Tatebe, Fukiko Kitani-Morii, Takuma Ohmichi, Yuzo Fujino, Kanako Menjo, Naoto Terada, Miho Osako, Yoko Mochizuki, Satoshi Teramukai, Takahiko Tokuda, Toshiki Mizuno, Takashi Kasai

**Affiliations:** 1 Department of Neurology, Kyoto Prefectural University of Medicine, Kyoto, Japan; 2 Department of Neurology, Tokyo Metropolitan Kita Medical and Rehabilitation Center for the Disabled, Tokyo, Japan; 3 Department of Genetic Medicine, Jikei University, Tokyo, Japan; 4 Department of Functional Brain Imaging, Institute for Quantum Medical Science, National Institutes for Quantum Science and Technology, Chiba, Japan; 5 Department of Molecular Pathobiology of Brain Diseases, Kyoto Prefectural University of Medicine, Kyoto, Japan; 6 Hananoki Medical Welfare Center, Kyoto, Japan; 7 Department of Biostatistics, Kyoto Prefectural University of Medicine, Kyoto, Japan; Niigata University, JAPAN

## Abstract

The Dementia Screening Questionnaire for Individuals with Intellectual Disabilities (DSQIID) is an appropriate screening tool for detecting dementia in Down’s syndrome patients. However, whether this questionnaire reflects the neuropsychiatric signs of biomarker-confirmed Alzheimer’s disease in DS (DS-AD) remains unknown. To address this issue, we compared the plasma phosphorylated tau (P181tau: p-tau) level of a representative AD biomarker with the total score and each sub-score of the DSQIID. The DSQIID was completed by 43 of the 56 individuals enrolled in the study. The DSQIID total scores tended to be positively associated with age, and some sub-scores increased in an age-dependent manner. DSQIID total scores and some sub-scores were also positively correlated with plasma p-tau levels, while all significant correlations disappeared after adjusting for age. Moreover, one sub-score appeared to have a significant negative correlation with plasma p-tau levels after adjusting for age. The DSQIID likely reflects age-associated behavioral changes in patients with DS. Meanwhile, their scores did not correlate with plasma p-tau after adjusting for age, suggesting that there might be room for improvement in the DSQIID for detecting DS-AD.

## Introduction

Down syndrome (DS), a trisomy of chromosome 21, is the most common chromosomal disorder. Due to improvements in the quality of care for children with DS, the average life expectancy of DS has increased dramatically [1]. Another medical issue has emerged along with the prolonged longevity of DS. In other words, Alzheimer’s diagnosed in adults with DS (DS-AD). Pathological brain changes of individuals with DS over the age of 40 are almost identical to those of patients with Alzheimer’s disease (AD), consisting of both senile plaques and neurofibrillary tangles composed of amyloid β (Aβ) and phosphorylated tau (p-tau), respectively [2]. That is mainly because trisomy 21 leads to the overproduction of APP (Amyloid β Precursor Protein) on chromosome 21, which is known as one of the causative genes for familial AD [3], as well as overexpression of *Dyrk1A* and *RCAN1*, also located on chromosome 21, which are both involved in tau hyperphosphorylation [4]. In fact, DS confers a strongly increased risk of dementia, such that approximately 70–80% of the DS population has dementia by the age of 60 years, although not all individuals develop dementia even by 70 years of age [5, 6]. Therefore, individuals with DS represent a highly enriched population for AD.

Considering recent developments in disease-modifying therapies for AD, it is logical to include adults with DS in prevention trial for the disease the near future [7]. Nowadays, DS-AD can be diagnosed even in the preclinical period by using positron emission tomography (PET) for amyloid and tau as well as by fluid biomarker changes of decreased Aβ42 and elevated p-tau in cerebrospinal fluid (CSF) at specialized medical institutions [8–10]. In particular, recent advances in the highly sensitive quantification of plasma p-tau elevation have led to a safe, objective, and accurate hospital-based diagnosis of DS-AD [11, 12]. However, because there are large individual differences in baseline cognitive function, language development, and symptoms of dementia in patients with DS [13],prehospital screening for DS-AD by caregivers at home has been quite challenging; therefore, early enrollment of patients with DS-AD in prevention trials is difficult.

The Dementia Screening Questionnaire for Individuals with Intellectual Disabilities (DSQIID) is an appropriate screening tool for dementia in DS [14]. This instrument, developed by Deb et al. in 2007, is an observer-rated questionnaire consisting of 53 questions [15]. This method is characterized by a unique scoring system that overcomes the floor effect (i.e., the difficulty in assessing the relative loss of ability in subjects with lower gains in ability) of other existing dementia assessment scores. The questionnaire was divided into three parts. The first asked about the best ability a person has or has had. In the second part accounting for 43 of the 53 question scores, if the problems exist from before (“always been the case”) or there are no problems (“dose not apply”), the sub-items are scored zero, and only new symptoms (“always but worse”) and worsening problems (“new symptom”) are scored [15]. The third part contains 10 of the 53 questions, all of which are comparative; for example, ‘speak (signs) less” and “seems generally more tired“. If a response is “yes,” one point is scored. In general, part 1 was not involved in scoring. As no weighting factor was assigned to each question, the sum of the points added in parts 2 and 3 was used as the total score for the test. This method has already been translated into many languages and has been widely used as a prehospital screening tool for DS-AD that is currently sought for the following two reasons. The first is its unique design to screen for dementia, regardless of individual developmental variety. The second advantage is the convenience of observer-rated scoring for caregivers, in which all inquiries can be completed without visiting the hospital. However, there is no guarantee that each sub-score of the DSQIID genuinely reflects the neuropsychiatric signs of DS-AD. Because the DSQIID was developed before the era of biomarker-based diagnosis of AD, the scores have been validated only by the clinical diagnosis of DS-AD but not by AD biomarker changes. Therefore, some of the subscores in the DSQIID might be simple results of non-AD neuropsychiatric problems in adult DS, such as age-dependent personality changes, depression, and adjustment disorders.

Considering the background above, we conducted this cross-sectional observational study comparing AD biomarker changes to the total score of the DSQIID and each sub-item score of the questionnaire to validate the usefulness of the DSQIID for the detection of DS-AD. As mentioned above, a major purpose of the early detection of DS-AD is to introduce early intervention by anti-amyloid therapy (such as lecanemab and donanemab) in a population with DS [16, 17]. The definition of Alzheimer’s disease has undergone major changes. The new diagnostic criteria proposed clearly indicate that AD should be defined biologically and not based on clinical syndromes [18]. Considering such rapid changes in the clinical circumstances surrounding DS and AD, we defined DS-AD as DS individuals with biomarker changes that suggest the presence of amyloid and tau pathology, and not as individuals with DS who simply show a decline in social life capacities. We chose the levels of p-tau phosphorylated at the threonine-181 residue as the AD biomarker change, which is a representative Core 1 biomarker to detect the initial stage of AD in the new diagnostic criteria [18], because elevation of p-tau181 in the CSF and plasma occurs solely in AD and not in other neurodegenerative disorders [19, 20]. Since it is not feasible to collect CSF from all patients with DS, we used plasma p-tau 181 levels as a marker of DS-AD in this study; elevation of plasma p-tau 181is also a specific phenomenon reflecting early AD pathology in the brain [18, 21], correlated linearly with CSF levels, and has been repeatedly reported in DS-AD [11, 12]. Note: though various p-tau molecular species have been proposed as the AD biomarker, “p-tau” means p-181 tau in the following description in this study unless otherwise mentioned.

## Material and methods

### Study design, ethics statement, and participant recruitment

This was a retrospective study of a population for which informed consent was obtained as described below. The study data were accessed on September 29, 2021. All data were fully anonymized prior to access. Written informed consent to obtain data from their medical records and biomarker data used in the research was obtained before participation from the nearest caregiver or, if possible, from participants with DS. The study was approved by the University Ethics Committee of Kyoto Prefectural University of Medicine (KPUM), Kyoto, Japan (RBMR-C-1226), and by the Research Ethics Committee of Tokyo Metropolitan Kita Medical and Rehabilitation Center for the Disabled (number: 2019–22). Informed consent was obtained from patients in the DS group when possible and from their nearest relatives. This study was designed and conducted in accordance with the Declaration of Helsinki. We enrolled 56 patients with DS (DS group) registered for DS in the KPUM, Hananoki Medical Welfare Center, and Tokyo Metropolitan Kita Medical and Rehabilitation Center for the Disabled between February 2013 and December 2020. To set a reference value for p-tau, 33 healthy controls (control group) were enrolled from another KPUM registry. Participants in the control group provided written informed consent prior to participation. The registry protocols were approved by the medical ethics committee at KPUM (approval number: ERB-G-12). Control participants were not selected from among the family members of the DS group.

### Plasma samples and clinical data collection

Plasma samples were obtained via venous puncture and 8 mL of blood was collected in EDTA-containing tubes. After collection, the plasma was separated by centrifugation for 10 min at 3,000 rpm and placed in polypropylene vials. Fresh samples obtained from the enrolled participants were immediately stored at -80°C until analysis. The DS group were completed by their carers [22]. For most participants, the DSQIID was completed after consultation with their parents. For participants whose parents died or were aged out, the DSQIID was completed by a sibling or staff member at their residential facility. If the DSQIID was evaluated more than once, the results of the questionnaire with the shortest lag time from blood collection were used. DSQIID results, examined before or more than two years after blood collection, were not used for the study. Part 1 of the DSQIID questionnaire, which contained written answers and a few simple questions aimed at estimating the best abilities of the patients, was not included in the analysis. The total score in Parts 2 and 3 and the sub-score of each item represented as zero (the problems existing from before or there are no problems) or one (new symptoms appearing or worsening existing problems) were used for the following analysis.

### Diagnosis of DS-AD

It is difficult to diagnose DS-AD based on certain criteria because the intellectual disability of individuals with DS varies greatly from person to person. General criteria for the diagnosis of dementia are not currently specified for people with intellectual disabilities. Studies on DS-AD have used a method in which the diagnosis of DS-AD is made through consensus meetings in a team consisting of dementia specialists, family members or caregivers, and nurses [23]. For example, in a previous study of DS cohorts in Barcelona, London, Kentucky, Cambridge, and Munich, clinical dementia status was determined individually for each participant at a Consensus Case Conference, where these discussions included at least two clinicians with longstanding expertise in evaluating dementia in DS and included a review of the medical and psychiatric history and findings from the neurological exam, interviews with caregivers or family members, and the participant’s performance in the neuropsychological evaluation, taking into consideration the participants’ baseline intelligence quotient, medical and psychiatric conditions, and any major life events [24]. This method conforms to the Consensus Recommendation proposed by Moran et al., which excludes the possibility of cognitive decline due to medical illnesses, environmental changes, or psychological stressors [25]. Based on the above situation in this field and the pathological finding that AD pathology rarely occurs in patients with DS at the age of 30 years or younger [26], in this study, we reviewed the medical records retrospectively to confirm that 1) participants were 30 years or older, 2) the family member or caregiver has complained of a persistent decline in acquired daily living skills for at least 6 months, 3) no factors could explain current symptoms in interviews with the patient and family regarding the psychological impact and changes in living environment which might cause depression or psychiatric symptoms, physical examination, blood and urine tests (not including AD biomarkers) and 4) no lesions on head imaging tests could explain the current symptoms (e.g. hemorrhage, tumor, or infarction). In consensus meetings involving two or more neurologists, a diagnosis of DS-AD was made if the above four criteria were met. (authors: TK, CT, MO, MS, FKM, TO).

### Measurement of p-tau and apolipoprotein E typing

The plasma p-tau concentration was measured using the single-molecule immunoarray (Simoa) method with reagents from a single lot using the Simoa P-tau181 assay on an HD-1 Simoa analyzer according to the manufacturer’s protocol (Quanterix, Lexington, MA, USA). All samples were analyzed in duplicate on one occasion.

The apolipoprotein E (ApoE) haplotype was determined by genotyping using the Invader assay or DNA microarray method undertaken by BML Inc. (Tokyo, Japan) or PreMedica Inc. (Tokyo, Japan), or phenotyping by isoelectric electrophoresis for plasma [27] undertaken by BML Inc. (Tokyo, Japan).

### Statistics

The–Mann Whitney U test was used to compare two independent groups. Fisher’s exact test was used to evaluate the significance of categorical variables. Univariate correlation analysis was performed using the Spearman’s rank correlation coefficient (Spearman’s test). The difference in the slopes of the regression lines was determined by the significance of the interaction term in the analysis of covariance (ANCOVA). (SPSS ver. 23, IBM Japan Ltd, Tokyo, Japan) Multiple regression analysis was performed with plasma phosphorylated tau concentration after log-transformed as the dependent variable and DSQIID sub-items and age as explanatory variables. SPSS ver. 23 (IBM Japan Ltd., Tokyo, Japan) was used for the multivariate analyses. The normality test for the residuals of multiple regression analysis was performed using the D’Agostino-Pearson omnibus normality test and the Shapiro-Wilk normality test. The above analyses were performed using the GraphPad Prism software (version 9.0; San Diego, CA, USA). The level of significance was set at P<0.05.

## Results

### Age dependent elevation of plasma p-tau in DS with and without apolipoprotein E allele

**Fig 1:**
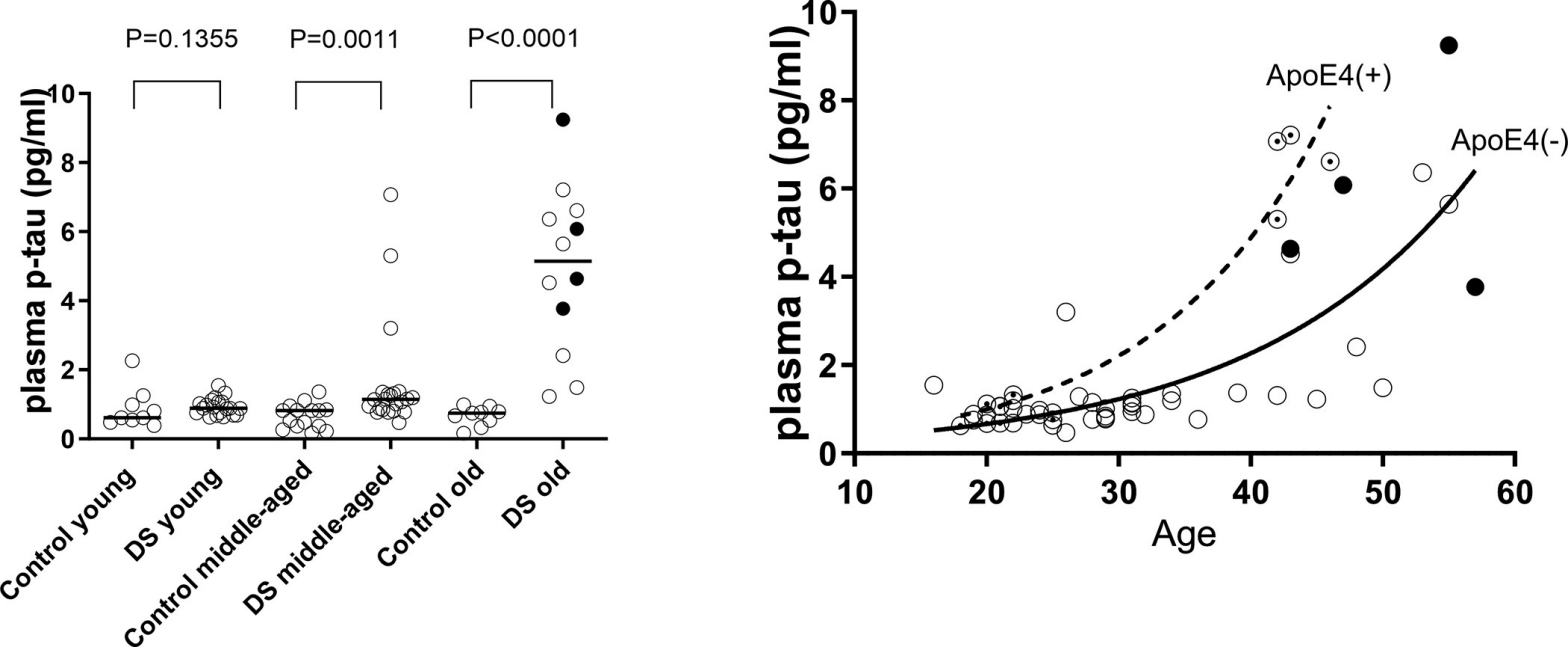
(A) Scatter plots of plasma p-tau levels in the DS and Control groups were shown. The participants were categorized by age into young into young (aged 16–26 years), middle-aged (aged 26–42 years), and old cases (aged older than 43 years). Black circles indicate DS-AD cases diagnosed according to the definitions given in the Method. Bars indicate median values. P values obtained by Man-Whitney’s U test were indicated in the columns. (B) Scatter plots regarding relationship between plasma p-tau level and age in the DS group were shown. Individuals with DS were dichotomized into ApoE4(+) (indicated by white circles with dots) and the ApoE4(-) (indicated by white or black circles) groups according to the presence of at least one ApoE4 allele. Black circles indicate DS-AD cases. At the time of plasma collection, there were no patients in the ApoE4(+) group diagnosed with DS-AD. The dashed line for the ApoE4(+) group and solid line for the ApoE4(-) group were regression curves.

As summarized in the upper part of Table 1 and S1 Table, no age or sex differences were observed between the DS and Control groups. None of the participants had chronic kidney disease or a history of stroke/myocardial infarction, which are major confounding factors for plasma p-tau measurement [28]. Apolipoprotein E (ApoE) genotypes or phenotypes were determined in 54 individuals among the 56 enrolled participants with DS, of which 3 had ApoE2/3, 40 had ApoE3/3, and 11 had ApoE3/4. No individual had ApoE2/2, ApoE2/4, or ApoE4/4 mutations. The allele frequencies of ApoE2, ApoE3, and ApoE4 were 2.8%, 87%, and 10.2% respectively. Four participants were diagnosed with DS-AD according to the definitions provided in the methods. The plasma p-tau levels were significantly higher in the DS group than in the control group. When the participants were categorized by age into young (aged16-25 years: n = 9 in control and n = 21 in DS), middle-aged (aged 26–42 years: n = 15 in control and n = 23 in DS), and old cases (over aged older than 43 years: n = 9 in control and n = 12 in DS) as followed the age categories used in previous biomarker studies in this cohort [29, 30], the difference of plasma p-tau between the groups was negligible in the young (P = 0.1355), became significant in the middle-aged (P = 0.0011), and was clearly observed with little overlap in the old cases (P = 0.0001) in Mann-Whitney’s U test (Fig 1A). As expected, plasma p-tau levels were strongly correlated with age in the DS group, whereas no significant relationship was observed in the control group using Spearman’s test (rs = 0.6725, P<0.0001 for the DS group and rs = -0.08301, P = 0.6460 for the control group).

**Table 1:** Demographics of the participants.

Participants with blood collection	DS group	Control group	Statistic value
Number of cases	56	33	
Age (mean ± SD)	31.66 ± 10.89	34.82 ± 10.82	*U* = 745, P = 0.1291 in Mann-Whitney’s U test
[maximum—minimum]	[57 – 16]	[56 – 14]	
Sex	Female:27, Male:29	Female:14, Male:19	P = 0.6630 in Fisher’s exact test
plasma p-tau (pg/ml) (mean±SD)	2.06 ± 2.11	0.72 ± 0.40	*U* = 336, P<0.0001 in Mann-Whitney’s U test
ApoE type			
E2/2	0	N/A	
E2/3	3	N/A	
E3/3	40	N/A	
E3/4	11	N/A	
E4/4	0	N/A	
N/A	2	N/A	
DS-AD	4	N/A	
Participants with DSQIID examined within 2 years of blood collection	DS group		
Number of cases	43		
Age (mean ± SD)	32.21 ± 10.79		
Sex	Female:21, Male:22		
plasma p-tau (pg/ml) (mean ± SD)	2.022 ± 2.20		
DSQIID total score (median (minimum-maximum)	9 (0–38)		
E2/2	0		
E2/3	1		
E3/3	31		
E3/4	9		
E4/4	0		
N/A	2		

SD: standard deviation, NA: not available

We dichotomized individuals with DS into ApoE4(+) and the ApoE4(-) groups according to the presence of at least one ApoE4 allele. No age or sex differences were found between these groups (P = 0.182 for age, P = 0.594 for sex in Mann-Whitney’s U test). As shown in Fig 1B, both groups showed significant age-dependent elevation of plasma p-tau levels in Spearman’s test (rs = 0.8153, P = 0.0015 for the ApoE4(+) group and rs = 0.6263, P<0.0001 for the ApoE4(-) group). We set the cutoff level for plasma p-tau at 1.52 pg/ml based on the calculation of the average value (0.72 pg/ml) + 2 × standard deviation (0.40 pg/ml) in the control group. Based on this cutoff, 12 participants with DS aged > 30 years, the youngest age at which amyloid pathology can be detected in DS [2], were identified to have abnormally elevated plasma p-tau levels. Furthermore, the plasma p-tau levels in four patients clinically diagnosed with DS-AD were well above the cutoff value. When focusing on the impact of the presence of the ApoE4 allele on such p-tau elevation in relatively younger ages of 40 to 50 years, four of the eight cases belonged to the ApoE4(+) group, while all four cases with abnormal p-tau values aged over 50 years were in the ApoE4(-) group. This promotive effect of the age-dependent elevation of p-tau by carrying the ApoE4 allele in DS was also suggested by the fact that the slope of the regression line between age and normalized (log-transformed) p-tau was significantly steeper in the ApoE4(+) group than in the ApoE4(-) group (the interaction term between age and group was significant: P<0.001 by ANCOVA). Note: Although no patients in the ApoE4(+) group were diagnosed with DS-AD at the time of plasma collection, four ApoE4(+) patients with elevated p-tau levels were confirmed to develop DS-AD within the next 2–3 years.

### Correlation analysis between DSQIID total / sub-item scores and plasma p-tau

We obtained valid responses to the DSQIID questionnaire from 43 individuals with DS among the 56 enrolled participants. The age, sex, total scores of DSQIID parts 2 and part3 (DSQIID total score), and levels of plasma p-tau in the participants are summarized in the lower part of Table 1 and S1 Table. The age-dependent elevation in p-tau levels remained statistically significant in these 43 individuals (rs = 0.7230, P<0.0001 by Spearman’s test). The DSQIID total scores tended to be positively associated with age; however, this association was not statistically significant (rs = 0.2652, P = 0.0857 by Spearman’s test).

We summarized the relationship between the scores (zero or one) of each sub-item of the DSQIID, age, and plasma p-tau levels (Table 2). Univariate analysis using Spearman’s test revealed that eight subitems of the DSQIID were significantly correlated with age. All these significant correlations were positive; that is, scoring on the sub-items was related to older age. Fourteen sub-items of the DSQIID significantly correlated with plasma p-tau levels. All these significant correlations were also positive; that is, scoring on the sub-items was related to higher p-tau. As expected from the strong positive correlation between age and p-tau level in the DS group, seven of the 14 questions positively correlated with p-tau levels and showed a significant correlation between score and age. However, these significant correlations between p-tau and the sub-item scores disappeared after adjusting for age in the multiple regression analysis. Similarly, as shown at the bottom of the table, there was a significant positive correlation between the DSQIID total scores and plasma p-tau levels in the univariate analysis using Spearman’s test, but this significant association disappeared after age adjustment. Interestingly, one sub-items, "Generally, appears more forgetful,” appeared significant negative correlations with plasma p-tau after age adjustment (P = 0.020, β = -0.224), i.e., scoring on this sub-item was related to lower p-tau suggesting absence of AD pathology.

**Table 2:** Correlation between sub-items of DSQIID, age, and plasma p-tau levels.

Sub-items of DSQIID	Univariate analysis for age		Univariate analysis for p-tau		Multivariate analysis for log p-tau value			
					each score		age	
	rs	P-value	rs	P-value	β	P-value	β	P-value
Part 2								
1. Cannot wash and/or bathe without help	0.053	0.735	0.269	0.081	0.120	0.215	**0.778**	**<0.001**
2. Cannot dress without help	0.256	0.098	**0.323**	**0.035**	0.017	0.866	**0.788**	**<0.001**
3. Dresses inappropriately (e.g. back to front, incomplete)	**0.374**	**0.014**	**0.352**	**0.021**	-0.034	0.750	**0.807**	**<0.001**
4. Undresses inappropriately (e.g. in public)	N/A	N/A	N/A	N/A	N/A	N/A	N/A	N/A
5. Needs help eating	**0.303**	**0.048**	**0.319**	**0.037**	0.120	0.249	**0.747**	**<0.001**
6. Needs help using the bathroom	0.23	0.138	0.223	0.15	-0.046	0.642	**0.805**	**<0.001**
7. Incontinent (including occasional accidents)	0.103	0.51	0.09	0.565	-0.059	0.541	**0.798**	**<0.001**
8. Does not initiate conversation	0.150	0.336	0.272	0.078	-0.013	0.898	**0.796**	**<0.001**
9. Cannot find words	0.166	0.293	**0.398**	**0.009**	0.049	0.623	**0.783**	**<0.001**
10. Cannot follow simple instructions	0.280	0.069	**0.351**	**0.021**	0.131	0.196	**0.751**	**<0.001**
11. Cannot follow more than one instruction at a time	**0.401**	**0.008**	**0.482**	**0.001**	-0.034	0.756	**0.809**	**<0.001**
12. Stops in the middle of a task	0.142	0.364	0.266	0.084	0.036	0.713	**0.788**	**<0.001**
13. Cannot read	0.191	0.221	0.299	0.052	0.048	0.632	**0.780**	**<0.001**
14. Cannot write (including printing own name)	0.17	0.275	**0.315**	**0.04**	0.115	0.243	**0.769**	**<0.001**
15. Changed sleep pattern (sleeping more or sleeping less)	-0.077	0.622	-0.013	0.936	0.100	0.298	**0.799**	**<0.001**
16. Wakes frequently at night	0.224	0.148	0.222	0.153	-0.032	0.751	**0.802**	**<0.001**
17. Confused at night	-0.181	0.247	-0.184	0.238	-0.028	0.777	**0.789**	**<0.001**
18. Sleeps during the day	0.035	0.823	-0.076	0.628	-0.109	0.258	**0.799**	**<0.001**
19. Wanders at night	-0.112	0.474	-0.236	0.127	-0.073	0.454	**0.786**	**<0.001**
20. Cannot find way in familiar surroundings	0.213	0.17	0.161	0.302	-0.031	0.753	**0.799**	**<0.001**
21. Wanders	N/A	N/A	N/A	N/A	N/A	N/A	N/A	N/A
22. Loses track of time (time of day, day of the week, seasons)	0.068	0.666	0.211	0.175	0.091	0.345	**0.786**	**<0.001**
23. Not confident walking over small cracks, lines on the ground or uneven surfaces	**0.368**	**0.015**	0.27	0.08	-0.026	0.802	**0.804**	**<0.001**
24. Unsteady walk, loses balance	0.214	0.169	0.251	0.104	0.106	0.290	**0.765**	**<0.001**
25. Cannot walk unaided	**0.321**	**0.036**	**0.302**	**0.049**	0.008	0.940	**0.791**	**<0.001**
26. Cannot recognize familiar person (staff / relatives)	-0.237	0.127	-0.187	0.231	0.032	0.749	**0.800**	**<0.001**
27. Cannot remember names of familiar persons	0.000	>0.999	0.228	0.141	0.145	0.128	**0.784**	**<0.001**
28. Cannot remember recent events	0.28	0.069	**0.31**	**0.043**	0.035	0.729	**0.783**	**<0.001**
29. Withdraws from social activities	-0.04	0.8	0.071	0.651	-0.025	0.797	**0.794**	**<0.001**
30. Withdraws from other persons	0.000	>0.9999	0.231	0.137	0.060	0.537	**0.792**	**<0.001**
31. Loss of interest in hobbies and activities	0.098	0.533	0.217	0.162	-0.043	0.659	**0.796**	**<0.001**
32. Seems to go into own world	0.076	0.628	0.115	0.462	-0.025	0.796	**0.794**	**<0.001**
33. Obsessive repetitive behaviour (e.g. empties cupboards repeatedly)	-0.043	0.784	0.09	0.565	-0.056	0.567	**0.787**	**<0.001**
34. Hides or hoards objects	0.162	0.299	0.11	0.481	-0.100	0.301	**0.801**	**<0.001**
35. Loses objects	0.187	0.229	0.14	0.37	-0.026	0.792	**0.797**	**<0.001**
36. Puts familiar things into wrong places	**0.413**	**0.006**	**0.424**	**0.005**	0.040	0.703	**0.779**	**<0.001**
37. Does not know what to do with familiar objects	**0.423**	**0.005**	**0.444**	**0.003**	0.186	0.080	**0.711**	**<0.001**
38. Appears insecure	0.139	0.4	0.14	0.37	-0.009	0.926	**0.795**	**<0.001**
39. Appears anxious or nervous	-0.141	0.368	0.06	0.703	0.052	0.594	**0.800**	**<0.001**
40. Appears depressed	0.133	0.397	0.271	0.079	0.072	0.456	**0.787**	**<0.001**
41. Shows aggression (verbal or physical)	-0.06	0.702	0.04	0.799	-0.013	0.897	**0.793**	**<0.001**
42. Fits / epilepsy	0.151	0.334	-0.059	0.708	-0.043	0.655	**0.798**	**<0.001**
43. Talks to self	0.188	0.227	0.159	0.309	-0.175	0.068	**0.821**	**<0.001**
Part 3.								
44. Lost some skills (e.g. brushing teeth)	0.178	0.253	0.212	0.172	0.032	0.745	**0.788**	**<0.001**
45. Speaks (or signs) less	-0.044	0.779	0.092	0.557	-0.005	0.960	**0.793**	**<0.001**
46. Seems generally more tired	0.017	0.912	0.1	0.525	-0.013	0.891	**0.795**	**<0.001**
47. Appears tearful, gets more easily upset	**0.355**	**0.02**	**0.408**	**0.007**	0.036	0.734	**0.780**	**<0.001**
48. Appears generally slower	0.18	0.247	**0.42**	**0.005**	0.041	0.679	**0.786**	**<0.001**
49. Slower speech	0.257	0.096	**0.33**	**0.031**	-0.054	0.585	**0.806**	**<0.001**
50. Appears more lazy	0.072	0.647	0.196	0.207	-0.016	0.871	**0.794**	**<0.001**
51. Walks slower	0.142	0.365	0.248	0.109	0.122	0.210	**0.771**	**<0.001**
52. Generally, appears more forgetful	0.253	0.101	0.094	0.549	**-0.224**	**0.020**	**0.847**	**<0.001**
53. Generally, appears more confused	0.199	0.201	0.263	0.088	-0.016	0.874	**0.797**	**<0.001**
DSQIID total score	0.265	0.086	**0.454**	**0.002**	0.180	0.862	**0.788**	**<0.001**

N/A: Not Applicable because no participant scored the relevant subitems.

β: standardized partial regression coefficient

Significant correlations between sub-item scores and p-tau levels are highlighted in bold font.

Note: Age-adjusted multivariate analysis was performed using multiple regression analysis. Significant models were obtained for all the analyses. No model was significantly different in the D’Agostino-Pearson omnibus or Shapiro-Wilk normality tests of the residuals.

According to the original DSQIID paper, Deb et al. conducted a forced four-factor analysis of the questions in Part 2 of the questionnaire and extracted the following four characteristics; 1. Memory / confusion, 2. Feelings of insecurity, 3. Sleep problems, 4. Behaviour problems [15]. We then sub-totaled the items scored in the questionnaire with respect to these four components and conducted univariate and multivariate analyses with age and p-tau levels in the same manner as above (Table 3). In this analysis, the scores of subcomponents 1 (memory/confusion) and 2 (feelings of insecurity) were correlated with plasma p-tau levels, but these correlations disappeared after age adjustment.

**Table 3:** Correlation between scores of sub-components of DSQIID part 2, age, and plasma p-tau levels.

Sub-components of DSQIID part 2	Univariate analysis for age		Univariate analysis for p-tau		Multivariate analysis for log p-tau value			
					each score		age	
	rs	P-value	rs	P-value	β	P-value	β	P-value
Component 1: Memory / confusion	**0.336**	**0.028**	**0.521**	**0.0003**	0.085	0.417	**0.762**	**<0.001**
Component 2: Feelings of insecurity	0.248	0.108	**0.446**	**0.003**	0.019	0.854	**0.788**	**<0.001**
Component 3: Sleep problems	0.093	0.553	0.074	0.637	-0.025	0.800	**0.797**	**<0.001**
Component 4: Behaviour problems	0.155	0.322	0.282	0.067	-0.041	0.674	**0.798**	**<0.001**

Scores on the 43 questions that comprise Part 2 of the DSQIID were categorized into the four components shown below. The scores for each component are the unweighted sum of the scores for the questions of the component.

Component 1: 1. Cannot wash and/or bathe without help, 2. Cannot dress without help; 3. Dresses inappropriately (e.g., back to front, incomplete), 9. Cannot find words; and 10. Cannot follow simple instructions. 11. Cannot follow more than one instruction at a time. 12. Stopping in the middle of the task, 13. Cannot read, 14. Cannot write (including printing their own names); 17. Confused at night, 20. Cannot find a way in familiar surroundings; 22. Time loss track (time of day, day of the week, and season), 23. Unconfident walking over small cracks, lines on the ground, or uneven surfaces; and 26. Cannot recognize a familiar person (staff member or relatives); 27. Cannot remember the names of familiar people, 28. Cannot remember recent events, 32. Seems to enter their own world: 37. Did not know what to do with familiar objects, and 43. Talks to self.

Component 2: 2. Cannot dress without help; and 5. Needs help eating, 6. Needs help using the bathroom. 7. incontinence (including occasional accidents), and 8. Does not initiate conversations; 24. Unsteady walk, loses balance, 25. Cannot walk unaided, 29. withdrawal from social activities and 30. Withdrawal from others, 31. Loss of interest in hobbies and activities, 38. Appears insecure, 39. Appear anxiety or nervousness; and 40. Appears depressed.

Component 3: 4. Undresses inappropriately (e.g., in public), 15. Changed sleep patterns (sleeping more or less); and 16. Wakes frequently at night; 18. Sleep during the day, 19. Wanders at night, 21. Wanders, and 42. Fits / epilepsy.

Component 4: 33. Obsessive repetitive behaviour (e.g., repeatedly emptiing cupboards), 34. Hides or hoarded objects: 35. Loses objects, 36. puts familiar things into incorrect places, and 41. shows aggression (verbal or physical) [15]

The study enrolled 30 (54%) individuals of relatively young adult Down syndrome younger than 30 years of age, who are generally assumed to have no amyloid pathology [2]. Furthermore, the usefulness of the DSQIID in relatively young patients has not yet been validated. Considering the fact that amyloid pathology in DS appears after the age of 30 years and that previous DSQIID validation studies have been conducted on DS participants over 20 or 40 years of age, the same analyses as those in Tables 2 and 3 were conducted on DS participants over 30 years of age. Although the number of question items and subcomponents for which significant differences were detected was reduced owing to the smaller number of participants in the analysis, similar results were observed in the analysis that included all participants. There was no positive significant correlation between sub-items / sub-components scores and plasma p-tau in multivariate analysis after age-adjustment, while two sub-items,” Talks to self" and "Generally, appears more forgetful,” appeared significant negative correlations with plasma p-tau after age-adjustment with reproducibility in participants aged 30 years and older. (P = 0.023, β = -0.339 and P = 0.047, β = -0.302, respectively) (S2A and S3 Tables). Another issue is the time lag between DSQIID and blood collection. In general, changes in plasma p-tau over time are as small as 4.5% per year [31], even in cases of dementia; therefore, the effect of this time lag on the current results is expected to be limited. However, the study included six cases in which the interval between the DSQIID and p-tau evaluation was > 1 year. The time lag between the DSQIID evaluation and blood collection in these six cases may not be negligible. Thus, we reanalyzed the data after excluding these six cases. The same results were obtained after the exclusion of these six cases. There was no positive significant correlation between sub-items / sub-components scores and plasma p-tau in multivariate analysis after age-adjustment, while two sub-items,” Talks to self" and "Generally, appears more forgetful,” appeared significant negative correlations with plasma p-tau after age-adjustment (P = 0.030, β = -0.491 and P = 0.049, β = -0.351, respectively) (S2B Table).

## Discussion

### The major findings of this study are as follows

First, plasma p-tau levels increased in an age-dependent manner, reaching an abnormal cutoff value in individuals over 40 years of age in the DS group. All the patients clinically diagnosed with DS-AD exhibited elevated plasma p-tau levels. These findings correspond with those of recent biomarker studies, including ours, and align with previous pathological and clinical observations that DS-AD typically becomes evident in patients in their 40s [2, 5, 6, 11, 12]. The finding that the slope of the regression line between age and p-tau was steeper in the ApoE4(+) group is also consistent with recent reports indicating that individuals with DS carrying the ApoE4 allele present with AD at younger ages than those without the ApoE4 allele [32]. Considering that elevated plasma p181-tau levels are specifically observed in MCI due to AD and the early phase of AD, but not in other types of neurodegenerative diseases such as vascular dementia or frontotemporal dementia [21, 33], these results validate our assumption that plasma p-tau concentrations can be used as a marker for detecting the development of DS-AD.

Second, the total scores of the DSQIID and the scores on 14 of the 53 sub-items were positively correlated with plasma levels of p-tau; however, none of these correlations remained significant after age adjustment. Considering the following two facts: seven of these 14 sub-item scores showing correlation with p-tau levels were also positively correlated with age, and DSQIID total scores tended to be positively associated with age, the total scores and some sub-scores in the DSQIID likely reflect, to some extent, age-related behavioral changes in individuals with DS. However, the lack of correlation between plasma p-tau and DSQIID total scores or any sub-items after age adjustment raises doubts about the previous assumption that DSQIID can effectively screen for DS-AD. Moreover, one sub-item, "Generally appears more forgetful," showed significant negative correlations with plasma p-tau after age adjustment in this study. However, it should be noted that this significant finding is only part of the exploratory analysis for hypothesis generation, as multiple analyses were conducted during the testing of each question item. Nevertheless, this unexpected result suggests that there is room for improvement in the DSQIID for detecting DS-AD. Although we cannot dismiss the potential confounding effect of the substantial number of young individuals with DS enrolled in this study, results similar to those obtained from participants of all ages were confirmed in the sub-analyses, from which DS participants younger than 30 years were excluded. In this study, we calculated the scores of the four sub-components in DSQIID part 2 and examined the age-adjusted correlations between these sub-component scores and p-tau levels; however, we could not identify any sub-components associated with elevated p-tau levels. Therefore, to enhance the ability of the DSQIID to effectively detect the development of DS-AD, we could not propose strategies for weighing specific subcomponents based on the results of this study. In the future, when revising this questionnaire for greater effectiveness, eliminating questions that are less relevant to DS-AD and incorporating symptoms that are more specific to DS-AD, such as myoclonus [34], may be considered.

We acknowledge that the small sample size was a significant limitation of this study, which may have weakened the statistical power to detect an association between the DSQIIS subscores and p-tau levels. Moreover, although plasma p-tau is the most reliable blood-based marker for detecting DS-AD, the lack of imaging biomarkers such as amyloid and tau PET is another limitation of this study. Future research will need to conduct large-scale case-control studies utilizing both fluid and imaging biomarkers to validate the relationship between DSQIID total and sub-item scores and DS-AD.

## Conclusion

We conducted comparisons between plasma p-tau of the blood-based biomarker for DS-AD and DSQIID total/sub-item scores, which is a structured caregiver interview specifically asking about functional and behavioral changes and is believed to be an appropriate screening tool for detecting dementia in DS. DSQIID total scores tended to be positively associated with age, and some subscores were significantly elevated, suggesting that DSQIID scores likely reflect behavioral changes associated with aging in individuals with DS. The DSQIID total scores and some sub-item scores were positively correlated with plasma p-tau elevation, but all these significant correlations disappeared after adjusting for age. Moreover, one sub-items, "Generally, appears more forgetful,” appeared significant negative correlations with plasma p-tau after age-adjustment. This lack of positive and even negative correlations between DSQIID sub-scores and p-tau after age adjustment led us to infer that there is room for improvement in DSQIID for the purpose of detecting DS-AD.

## Supporting information

S1 Table (XLSX)

S2 Table **A.** Correlation between sub-items of DSQIID, age, and plasma p-tau levels in DS individuals aged more than 30 years. **B.** Correlation between sub-items of DSQIID, age, and plasma p-tau levels in DS individuals whose blood collection were conducted within 12 months of DSAQIID evaluation.
(DOCX)

S3 Table Correlation between scores of sub-components of DSQIID part 2, age, and plasma p-tau levels in DS individuals aged more than 30 years. (DOCX)
