# Supplementary material for: Comparison between DSQIID total / sub-item scores and plasma p-tau elevation in adults with Down’s syndrome
Source: PLoS One. 2024 Dec 9;19(12):e0311878. doi: 10.1371/journal.pone.0311878 (PMC11627409; doi:10.1371/journal.pone.0311878)
Supplement: S2 Table — A. Correlation between sub-items of DSQIID, age, and plasma p-tau levels in DS individuals aged more than 30 years. B. Correlation between sub-items of DSQIID, age, and plasma p-tau levels in DS individuals whose blood collection were conducted within 12 months of DSAQIID evaluation. (DOCX) [file pone.0311878.s002.docx]

**Supplementary Table 2A** Correlation between sub-items of DSQIID, age, and plasma p-tau levels in DS individuals aged more than 30 years

| Sub-items of DSQIID | Univariate analysis for age | | Univariate analysis for p-tau | | Multivariate analysis for p-tau | | | |
| --- | --- | --- | --- | --- | --- | --- | --- | --- |
|  |  |  |  |  | each score | | age | |
|  | rs | P-value | rs | P-value | β | P-value | β | P-value |
| Part 2 |  |  |  |  |  |  |  |  |
| 1. Cannot wash and/or bathe without help | **0.456** | **0.038** | 0.418 | 0.06 | 0.058 | 0.741 | **0.717** | **0.001** |
| 2. Cannot dress without help | 0.400 | 0.072 | 0.295 | 0.194 | -0.106 | 0.555 | **0.789** | **<0.001** |
| 3. Dresses inappropriately (e.g. back to front, incomplete) | **0.521** | **0.016** | 0.369 | 0.1 | -0.137 | 0.461 | **0.811** | **<0.001** |
| 4. Undresses inappropriately (e.g. in public) | N/A | N/A | N/A | N/A | #N/A | #N/A | #N/A | #N/A |
| 5. Needs help eating | **0.575** | **0.006** | 0.421 | 0.058 | 0.110 | 0.580 | **0.676** | **0.003** |
| 6. Needs help using the bathroom | -0.008 | 0.972 | 0.016 | 0.945 | -0.050 | 0.757 | **0.741** | **<0.001** |
| 7. Incontinent (including occasional accidents) | 0.000 | 1.000 | -0.122 | 0.599 | -0.136 | 0.392 | **0.741** | **<0.001** |
| 8. Does not initiate conversation | 0.147 | 0.525 | 0.081 | 0.727 | -0.116 | 0.472 | **0.755** | **<0.001** |
| 9. Cannot find words | 0.279 | 0.221 | 0.203 | 0.377 | -0.176 | 0.282 | **0.787** | **<0.001** |
| 10. Cannot follow simple instructions | 0.237 | 0.302 | 0.373 | 0.096 | 0.189 | 0.249 | **0.688** | **<0.001** |
| 11. Cannot follow more than one instruction at a time | **0.456** | **0.038** | 0.334 | 0.139 | -0.176 | 0.313 | **0.816** | **<0.001** |
| 12. Stops in the middle of a task | 0.219 | 0.34 | 0.192 | 0.406 | -0.025 | 0.878 | **0.746** | **<0.001** |
| 13. Cannot read | **0.437** | **0.048** | 0.295 | 0.194 | -0.097 | 0.586 | **0.784** | **<0.001** |
| 14. Cannot write (including printing own name) | 0.303 | 0.182 | 0.341 | 0.131 | 0.117 | 0.484 | **0.707** | **<0.001** |
| 15. Changed sleep pattern (sleeping more or sleeping less) | 0.186 | 0.419 | 0.332 | 0.141 | 0.181 | 0.257 | **0.715** | **<0.001** |
| 16. Wakes frequently at night | 0.289 | 0.203 | 0.244 | 0.287 | -0.066 | 0.696 | **0.761** | **<0.001** |
| 17. Confused at night | N/A | N/A | N/A | N/A | #N/A | #N/A | #N/A | #N/A |
| 18. Sleeps during the day | 0.176 | 0.447 | 0.054 | 0.818 | -0.143 | 0.376 | **0.767** | **<0.001** |
| 19. Wanders at night | N/A | N/A | N/A | N/A | #N/A | #N/A | #N/A | #N/A |
| 20. Cannot find way in familiar surroundings | 0.000 | 1.000 | 0.112 | 0.628 | 0.017 | 0.918 | **0.741** | **<0.001** |
| 21. Wanders | N/A | N/A | N/A | N/A | #N/A | #N/A | #N/A | #N/A |
| 22. Loses track of time (time of day, day of the week, seasons) | 0.01 | 0.965 | 0.18 | 0.434 | 0.057 | 0.721 | **0.739** | **<0.001** |
| 23. Not confident walking over small cracks, lines on the ground or uneven surfaces | **0.464** | **0.034** | 0.3 | 0.186 | -0.062 | 0.734 | **0.769** | **<0.001** |
| 24. Unsteady walk, loses balance | **0.453** | **0.039** | 0.36 | 0.109 | 0.111 | 0.535 | **0.691** | **0.001** |
| 25. Cannot walk unaided | 0.283 | 0.214 | 0.202 | 0.379 | -0.030 | 0.860 | **0.749** | **<0.001** |
| 26. Cannot recognize familiar person (staff / relatives) | N/A | N/A | N/A | N/A | #N/A | #N/A | #N/A | #N/A |
| 27. Cannot remember names of familiar persons | 0.204 | 0.376 | 0.225 | 0.327 | 0.033 | 0.842 | **0.734** | **<0.001** |
| 28. Cannot remember recent events | 0.298 | 0.19 | 0.277 | 0.224 | -0.005 | 0.978 | **0.742** | **<0.001** |
| 29. Withdraws from social activities | 0.214 | 0.352 | 0.037 | 0.874 | -0.164 | 0.307 | **0.771** | **<0.001** |
| 30. Withdraws from other persons | 0.149 | 0.52 | 0.055 | 0.812 | -0.113 | 0.483 | **0.755** | **<0.001** |
| 31. Loss of interest in hobbies and activities | 0.065 | 0.779 | 0.037 | 0.874 | -0.146 | 0.358 | **0.742** | **<0.001** |
| 32. Seems to go into own world | -0.071 | 0.761 | 0.02 | 0.931 | -0.009 | 0.958 | **0.740** | **<0.001** |
| 33. Obsessive repetitive behaviour (e.g. empties cupboards repeatedly) | -0.233 | 0.31 | -0.185 | 0.423 | -0.193 | 0.240 | **0.687** | **<0.001** |
| 34. Hides or hoards objects | -0.122 | 0.6 | -0.134 | 0.563 | -0.148 | 0.355 | **0.723** | **<0.001** |
| 35. Loses objects | -0.045 | 0.846 | 0.067 | 0.772 | 0.025 | 0.877 | **0.742** | **<0.001** |
| 36. Puts familiar things into wrong places | 0.065 | 0.779 | 0.34 | 0.132 | 0.099 | 0.536 | **0.737** | **<0.001** |
| 37. Does not know what to do with familiar objects | 0.175 | 0.447 | 0.383 | 0.087 | 0.201 | 0.214 | **0.693** | **<0.001** |
| 38. Appears insecure | -0.081 | 0.728 | 0.000 | 1.000 | -0.016 | 0.923 | **0.739** | **<0.001** |
| 39. Appears anxious or nervous | 0.125 | 0.591 | 0.067 | 0.772 | -0.093 | 0.560 | **0.750** | **<0.001** |
| 40. Appears depressed | -0.102 | 0.66 | 0.09 | 0.698 | 0.103 | 0.523 | **0.755** | **<0.001** |
| 41. Shows aggression (verbal or physical) | 0.01 | 0.965 | -0.04 | 0.863 | -0.081 | 0.615 | **0.741** | **<0.001** |
| 42. Fits / epilepsy | 0.054 | 0.816 | -0.107 | 0.644 | -0.013 | 0.936 | **0.741** | **<0.001** |
| 43. Talks to self | 0.053 | 0.821 | -0.139 | 0.547 | **-0.339** | **0.023** | **0.738** | **<0.001** |
| Part 3. |  |  |  |  |  |  |  |  |
| 44. Lost some skills (e.g. brushing teeth) | 0.147 | 0.525 | 0.178 | 0.44 | 0.045 | 0.784 | **0.734** | **<0.001** |
| 45. Speaks (or signs) less | -0.082 | 0.725 | -0.162 | 0.483 | -0.094 | 0.559 | **0.727** | **<0.001** |
| 46. Seems generally more tired | 0.245 | 0.285 | 0.13 | 0.576 | -0.074 | 0.657 | **0.760** | **<0.001** |
| 47. Appears tearful, gets more easily upset | 0.245 | 0.285 | 0.308 | 0.175 | 0.016 | 0.922 | **0.736** | **<0.001** |
| 48. Appears generally slower | 0.192 | 0.404 | 0.095 | 0.681 | -0.161 | 0.316 | **0.765** | **<0.001** |
| 49. Slower speech | 0.126 | 0.586 | 0.067 | 0.774 | -0.151 | 0.345 | **0.758** | **<0.001** |
| 50. Appears more lazy | -0.304 | 0.18 | -0.318 | 0.16 | -0.159 | 0.339 | **0.693** | **<0.001** |
| 51. Walks slower | 0.152 | 0.51 | 0.318 | 0.16 | 0.174 | 0.278 | **0.708** | **<0.001** |
| 52. Generally, appears more forgetful | -0.024 | 0.918 | -0.143 | 0.536 | **-0.302** | **0.047** | **0.735** | **<0.001** |
| 53. Generally, appears more confused | 0.109 | 0.637 | 0.15 | 0.516 | -0.039 | 0.810 | **0.745** | **<0.001** |
| DSQIID total score | 0.325 | 0.151 | 0.297 | 0.191 | -0.093 | 0.579 | **0.768** | **<0.001** |

N/A：Not Applicable because no participants were scored for the relevant sub-items.

β：standardized partial regression coefficient

Significant correlations between the scores of sub-items and p-tau levels were highlighted in bold.

Note: Age-adjusted multivariate analysis was performed using multiple regression analysis. Significant models were obtained for all analyses. No model was significantly different in the D'Agostino-Pearson omnibus normality test or Shapiro-Wilk normality test of residuals.

**Supplementary Table 2B** Correlation between sub-items of DSQIID, age, and plasma p-tau levels in DS individuals whose blood collection were conducted within 12 months of DSAQIID evaluation.

| Sub-items of DSQIID | Univariate analysis for age | | Univariate analysis for p-tau | | Multivariate analysis for p-tau | | | |
| --- | --- | --- | --- | --- | --- | --- | --- | --- |
|  |  |  |  |  | each score | | age | |
|  | rs | P-value | rs | P-value | β | P-value | β | P-value |
| Part 2 |  |  |  |  |  |  |  |  |
| 1. Cannot wash and/or bathe without help | 0.163 | 0.336 | 0.325 | 0.050 | 0.146 | 0.451 | **0.055** | **<0.001** |
| 2. Cannot dress without help | 0.306 | 0.066 | 0.282 | 0.091 | -0.185 | 0.457 | **0.059** | **<0.001** |
| 3. Dresses inappropriately (e.g. back to front, incomplete) | **0.344** | **0.037** | 0.323 | 0.051 | -0.205 | 0.415 | **0.059** | **<0.001** |
| 4. Undresses inappropriately (e.g. in public) | N/A | N/A | N/A | N/A | N/A | N/A | N/A | N/A |
| 5. Needs help eating | **0.388** | **0.018** | 0.269 | 0.107 | 0.030 | 0.923 | **0.056** | **<0.001** |
| 6. Needs help using the bathroom | 0.301 | 0.070 | 0.295 | 0.076 | -0.156 | 0.445 | **0.058** | **<0.001** |
| 7. Incontinent (including occasional accidents) | 0.219 | 0.194 | 0.271 | 0.105 | -0.071 | 0.739 | **0.057** | **<0.001** |
| 8. Does not initiate conversation | 0.183 | 0.279 | 0.318 | 0.055 | 0.046 | 0.794 | **0.056** | **<0.001** |
| 9. Cannot find words | 0.282 | 0.091 | **0.453** | **0.005** | 0.081 | 0.737 | **0.056** | **<0.001** |
| 10. Cannot follow simple instructions | **0.402** | **0.014** | **0.382** | **0.020** | 0.264 | 0.192 | **0.052** | **<0.001** |
| 11. Cannot follow more than one instruction at a time | **0.474** | **0.003** | **0.465** | **0.004** | -0.186 | 0.376 | **0.060** | **<0.001** |
| 12. Stops in the middle of a task | 0.174 | 0.302 | 0.207 | 0.220 | -0.052 | 0.797 | **0.057** | **<0.001** |
| 13. Cannot read | 0.230 | 0.170 | 0.234 | 0.164 | -0.096 | 0.692 | **0.057** | **<0.001** |
| 14. Cannot write (including printing own name) | 0.215 | 0.201 | 0.259 | 0.121 | 0.124 | 0.624 | **0.056** | **<0.001** |
| 15. Changed sleep pattern (sleeping more or sleeping less) | -0.097 | 0.568 | -0.125 | 0.460 | 0.051 | 0.792 | **0.057** | **<0.001** |
| 16. Wakes frequently at night | 0.183 | 0.278 | 0.165 | 0.329 | -0.135 | 0.504 | **0.058** | **<0.001** |
| 17. Confused at night | -0.101 | 0.552 | -0.280 | 0.093 | -0.372 | 0.323 | **0.055** | **<0.001** |
| 18. Sleeps during the day | 0.033 | 0.845 | -0.081 | 0.632 | -0.252 | 0.309 | **0.057** | **<0.001** |
| 19. Wanders at night | -0.125 | 0.461 | -0.265 | 0.112 | -0.365 | 0.488 | **0.056** | **<0.001** |
| 20. Cannot find way in familiar surroundings | 0.249 | 0.137 | 0.179 | 0.288 | -0.061 | 0.828 | **0.057** | **<0.001** |
| 21. Wanders | N/A | N/A | N/A | N/A | N/A | N/A | N/A | N/A |
| 22. Loses track of time (time of day, day of the week, seasons) | 0.171 | 0.313 | 0.222 | 0.186 | 0.173 | 0.493 | **0.055** | **<0.001** |
| 23. Not confident walking over small cracks, lines on the ground or uneven surfaces | 0.347 | 0.035 | 0.220 | 0.191 | -0.274 | 0.356 | **0.060** | **<0.001** |
| 24. Unsteady walk, loses balance | 0.288 | 0.084 | 0.176 | 0.297 | -0.023 | 0.945 | **0.057** | **<0.001** |
| 25. Cannot walk unaided | 0.275 | 0.100 | 0.224 | 0.183 | -0.279 | 0.478 | **0.058** | **<0.001** |
| 26. Cannot recognize familiar person (staff / relatives) | -0.266 | 0.112 | -0.203 | 0.228 | 0.167 | 0.756 | **0.057** | **<0.001** |
| 27. Cannot remember names of familiar persons | 0.000 | >0.999 | 0.252 | 0.133 | 0.420 | 0.085 | **0.056** | **<0.001** |
| 28. Cannot remember recent events | **0.360** | **0.029** | 0.252 | 0.133 | -0.154 | 0.561 | **0.058** | **<0.001** |
| 29. Withdraws from social activities | -0.051 | 0.763 | -0.023 | 0.893 | -0.182 | 0.340 | **0.056** | **<0.001** |
| 30. Withdraws from other persons | 0.080 | 0.639 | 0.248 | 0.139 | 0.097 | 0.624 | **0.056** | **<0.001** |
| 31. Loss of interest in hobbies and activities | 0.192 | 0.255 | 0.236 | 0.160 | -0.141 | 0.479 | **0.057** | **<0.001** |
| 32. Seems to go into own world | 0.080 | 0.638 | 0.123 | 0.468 | -0.021 | 0.919 | **0.056** | **<0.001** |
| 33. Obsessive repetitive behaviour (e.g. empties cupboards repeatedly) | -0.063 | 0.712 | 0.103 | 0.546 | -0.082 | 0.672 | **0.056** | **<0.001** |
| 34. Hides or hoards objects | 0.185 | 0.273 | 0.123 | 0.468 | -0.447 | 0.234 | **0.058** | **<0.001** |
| 35. Loses objects | 0.212 | 0.207 | 0.155 | 0.360 | -0.022 | 0.936 | **0.056** | **<0.001** |
| 36. Puts familiar things into wrong places | **0.464** | **0.004** | **0.472** | **0.003** | 0.142 | 0.509 | **0.054** | **<0.001** |
| 37. Does not know what to do with familiar objects | **0.468** | **0.004** | **0.495** | **0.002** | 0.486 | 0.061 | **0.048** | **<0.001** |
| 38. Appears insecure | 0.148 | 0.381 | 0.156 | 0.358 | -0.027 | 0.914 | **0.056** | **<0.001** |
| 39. Appears anxious or nervous | -0.080 | 0.638 | 0.055 | 0.745 | 0.034 | 0.870 | **0.056** | **<0.001** |
| 40. Appears depressed | 0.046 | 0.785 | 0.195 | 0.248 | 0.046 | 0.882 | **0.056** | **<0.001** |
| 41. Shows aggression (verbal or physical) | -0.060 | 0.725 | 0.057 | 0.738 | 0.008 | 0.967 | **0.056** | **<0.001** |
| 42. Fits / epilepsy | 0.172 | 0.309 | -0.056 | 0.744 | -0.162 | 0.605 | **0.057** | **<0.001** |
| 43. Talks to self | 0.217 | 0.198 | 0.103 | 0.544 | **-0.491** | **0.030** | **0.059** | **<0.001** |
| Part 3. |  |  |  |  |  |  |  |  |
| 44. Lost some skills (e.g. brushing teeth) | 0.191 | 0.257 | 0.150 | 0.377 | -0.033 | 0.863 | **0.057** | **<0.001** |
| 45. Speaks (or signs) less | 0.018 | 0.916 | 0.102 | 0.547 | -0.020 * | 0.907 * | **0.056*** | **<0.001*** |
| 46. Seems generally more tired | 0.107 | 0.528 | 0.131 | 0.441 | -0.114 | 0.520 | **0.057** | **<0.001** |
| 47. Appears tearful, gets more easily upset | **0.331** | **0.045** | 0.393 | 0.016 | 0.000 | 0.999 | **0.056** | **<0.001** |
| 48. Appears generally slower | 0.122 | 0.472 | **0.351** | **0.033** | 0.035 | 0.840 | **0.056** | **<0.001** |
| 49. Slower speech | 0.284 | 0.089 | 0.319 | 0.055 | -0.092 | 0.651 | **0.057** | **<0.001** |
| 50. Appears more lazy | 0.086 | 0.612 | 0.253 | 0.131 | 0.025 | 0.881 | **0.056** | **<0.001** |
| 51. Walks slower | 0.146 | 0.389 | 0.196 | 0.245 | 0.158 * | 0.380 * | **0.055 *** | **<0.001*** |
| 52. Generally, appears more forgetful | 0.292 | 0.079 | 0.117 | 0.492 | **-0.351** | **0.049** | **0.060** | **<0.001** |
| 53. Generally, appears more confused | 0.231 | 0.169 | 0.217 | 0.198 | -0.166 | 0.395 | **0.058** | **<0.001** |
| DSQIID total score | 0.325 | 0.050 | **0.438** | **0.007** | -0.003 | 0.741 | **0.057** | **<0.001** |

N/A：Not Applicable because no participants were scored for the relevant sub-items.

β：standardized partial regression coefficient

Significant correlations between the scores of sub-items and p-tau levels were highlighted in bold.

Note: Age-adjusted multivariate analysis was performed using multiple regression analysis. Significant models were obtained for all analyses. Boxes with asterisks (Q45 and Q51) indicate that there was significant difference in the D'Agostino-Pearson omnibus normality test or Shapiro-Wilk normality test of residuals, therefore validity of multivariate analysis is not ensured in those boxes.
