# Supplementary material for: Comparison between DSQIID total / sub-item scores and plasma p-tau elevation in adults with Down’s syndrome
Source: PLoS One. 2024 Dec 9;19(12):e0311878. doi: 10.1371/journal.pone.0311878 (PMC11627409; doi:10.1371/journal.pone.0311878)
Supplement: S3 Table — (DOCX) [file pone.0311878.s003.docx]

**Supplementary Table 3** Correlation between scores of sub-components of DSQIID part 2, age, and plasma p-tau levels in DS individuals aged more than 30 years

| Sub-components of DSQIID part 2 | Univariate analysis for age | | Univariate analysis for p-tau | | Multivariate analysis for p-tau | | | |
| --- | --- | --- | --- | --- | --- | --- | --- | --- |
|  |  |  |  |  | each score | | age | |
|  | rs | P-value | rs | P-value | β | P-value | β | P-value |
| Component 1: Memory / confusion | 0.3890 | 0.0813 | **0.5212** | **0.0154** | 0.022 | 0.901 | **0.732** | **0.001** |
| Component 2: Feelings of insecurity | 0.2771 | 0.2240 | 0.2836 | 0.2129 | -0.123 | 0.466 | **0.782** | **<0.001** |
| Component 3: Sleep problems | 0.3263 | 0.1488 | 0.2865 | 0.2079 | 0.005 | 0.977 | **0.739** | **<0.001** |
| Component 4: Behaviour problems | -0.0504 | 0.8280 | 0.1065 | 0.6458 | -0.071 | 0.658 | **0.733** | **<0.001** |

Scores on the 43 questions that comprise Part 2 of the DSQIID score were categorized into the four components shown below. The scores for each component are the unweighted sum of the scores on the questions of the component.

Component 1: 1. Cannot wash and/or bathe without help, 2. Cannot dress without help, 3. Dresses inappropriately (e.g. back to front, incomplete), 9. Cannot find words, 10. Cannot follow simple instructions, 11. Cannot follow more than one instruction at a time, 12. Stops in the middle of a task, 13. Cannot read, 14. Cannot write (including printing own name), 17. Confused at night, 20. Cannot find way in familiar surroundings, 22. Loses track of time (time of day, day of the week, seasons), 23. Not confident walking over small cracks, lines on the ground or uneven surfaces, 26. Cannot recognize familiar person (staff / relatives), 27. Cannot remember names of familiar persons, 28. Cannot remember recent events, 32. Seems to go into own world, 37. Does not know what to do with familiar objects, and 43. Talks to self.

Component 2: 2. Cannot dress without help, 5. Needs help eating, 6. Needs help using the bathroom, 7. Incontinent (including occasional accidents), 8. Does not initiate conversation, 24. Unsteady walk, loses balance, 25. Cannot walk unaided, 29. Withdraws from social activities, 30. Withdraws from other persons, 31. Loss of interest in hobbies and activities, 38. Appears insecure, 39. Appears anxious or nervous, and 40. Appears depressed.

Component 3: 4. Undresses inappropriately (e.g. in public), 15. Changed sleep pattern (sleeping more or sleeping less), 16. Wakes frequently at night, 18. Sleeps during the day, 19. Wanders at night, 21. Wanders, and 42. Fits / epilepsy.

Component 4: 33. Obsessive repetitive behaviour (e.g. empties cupboards repeatedly), 34. Hides or hoards objects, 35. Loses objects, 36. Puts familiar things into wrong places, and 41. Shows aggression (verbal or physical).
